# The Impact of the Human DNA Topoisomerase II C-Terminal Domain on Activity

**DOI:** 10.1371/journal.pone.0001754

**Published:** 2008-03-12

**Authors:** Emma L. Meczes, Kathryn L. Gilroy, Katherine L. West, Caroline A. Austin

**Affiliations:** 1 Institute for Cell and Molecular Biosciences, The University of Newcastle upon Tyne, Newcastle Upon Tyne, United Kingdom; 2 Division of Cancer Sciences and Molecular Pathology, University of Glasgow, Glasgow, United Kingdom; Temasek Life Sciences Laboratory, Singapore

## Abstract

**Background:**

Type II DNA topoisomerases (topos) are essential enzymes needed for the resolution of topological problems that occur during DNA metabolic processes. Topos carry out an ATP-dependent strand passage reaction whereby one double helix is passed through a transient break in another. Humans have two topoII isoforms, α and β, which while enzymatically similar are differentially expressed and regulated, and are thought to have different cellular roles. The C-terminal domain (CTD) of the enzyme has the most diversity, and has been implicated in regulation. We sought to investigate the impact of the CTD domain on activity.

**Methodology/Principle Findings:**

We have investigated the role of the human topoII C-terminal domain by creating constructs encoding C-terminally truncated recombinant topoIIα and β and topoIIα+β-tail and topoIIβ+α-tail chimeric proteins. We then investigated function *in vivo* in a yeast system, and *in vitro* in activity assays. We find that the C-terminal domain of human topoII isoforms is needed for *in vivo* function of the enzyme, but not needed for cleavage activity. C-terminally truncated enzymes had similar strand passage activity to full length enzymes, but the presence of the opposite C-terminal domain had a large effect, with the topoIIα-CTD increasing activity, and the topoIIβ-CTD decreasing activity.

**Conclusions/Significance:**

*In vivo* complementation data show that the topoIIα C-terminal domain is needed for growth, but the topoIIβ isoform is able to support low levels of growth without a C-terminal domain. This may indicate that topoIIβ has an additional localisation signal. *In vitro* data suggest that, while the lack of any C-terminal domain has little effect on activity, the presence of either the topoIIα or β C-terminal domain can affect strand passage activity. Data indicates that the topoIIβ-CTD may be a negative regulator. This is the first report of *in vitro* data with chimeric human topoIIs.

## Introduction

Type II DNA topoisomerases (topos II) are essential enzymes that resolve topological problems with DNA that arise during processes such as DNA replication and transcription, and chromosome segregation. Their mechanism involves the passing of one DNA duplex through a transient covalent break in a second duplex, in an ATP-dependent reaction. Humans have two topoisomerase II isoforms, α and β, which, while similar, have distinct patterns of expression and are thought to have different cellular roles [1]. Human topoIIα is thought to be the isoform primarily involved with DNA replication and chromosome segregation, while human topoIIβ has recently been implicated in transcriptional regulation [2]–[4].

While topoII isoforms show a high degree of sequence homology, approximately 70% between human topoII α and β, this is mainly in the N-terminal three-quarters of the protein sequence where the two catalytic centres are located. The C-terminal quarter of the protein, while always highly charged, shows much more sequence diversity. The C-terminal domain has been shown to be vital for cell viability. However, as C-terminal truncations are active *in vitro*, the essential nature of the C-terminal domain is thought to be linked to regulation [5].

Phosphorylation is a major form of regulation of human topoIIs, and has been shown to affect activity. Most modification sites are in the C-terminal domain, although a modification site at human topoIIα residue S29 that is a substrate for protein kinase C has also been identified [6]. Phosphorylation sites have been identified in *S. cerevisiae* topoII [7]. Some of these modifications are cell cycle specific, with modification at S1354, S1357, S1364 and T1366 being increased during mitosis, and modification at positions T1259, S1273, S1270 and S1267 increasing in G1 [7].

Considering the human enzymes, phosphorylation sites have been identified in topoIIα, with casein kinase II (CKII) being a principle kinase. αS1524 was identified as a principle phosphorylation site [8]. Several studies have linked human topoIIα phosphorylation to events at mitosis, and phosphorylation of αS1212 has been shown to occur at only at mitosis [9]. Phosphorylation has been suggested to activate topoII for chromatid segregation in anaphase [10], and CKII mediated αS1469 phosphorylation has been linked to chromatin condensation at prophase [11]. Additionally, αT1342 has been proposed to regulate mitotic functions [12], although another study has shown this to be phosphorylated throughout the cell cycle [13]. Human topoisomerase II phosphorylation sites, both cell cycle dependent and independent, are reviewed in Austin and Marsh, 1998 [5].

Modification by SUMO (small ubiquitin-like modifier) has also been linked to topoII regulation, with work in *S. cerevisiae* indicating that the topoII major modification sites (K1220, K1246/K1247 and K1277/K1278) again lie in the C-terminal domain [14]. In *S. cerevisiae* topoII, SUMO modification has been linked to chromosome stability, with modified topoII enriched at centromeric regions [15]. Additionally, topoII was found to be SUMO modified at metaphase, and was proposed to be essential for centromeric cohesion [14].

Work with human topo enzymes has shown that SUMO is rapidly conjugated to topoI, topoIIα and topoIIβ in response to DNA damage [16]–[17]. Additionally, after exposure to topoisomerase II inhibitor ICRF-193 human topoIIβ, but not topoIIα was selectively degraded by the proteasome, an activity that was abolished when the SUMO conjugating enzyme Ubc9 was knocked out. This implies that the degradation was linked to SUMO modification, and that this modification differs between the topoIIα and topoIIβ isoforms [18].

A major regulatory feature found in the C-terminal domains of topoIIs are nuclear localisation and nuclear export sequences (NLSs and NESs respectively). Without these signals, the enzyme is not able to localise to the nucleus, where it is essential during DNA replication, and cell viability is thus diminished or lost. Considering the human topoII isoforms, in topoIIα a strong NLS is found at 1454–1497 [19]–[20], and consistent with this, a mutant lacking residues 1490–1492 is unable to locate to the nucleus [21]. Another moderate NLS has been found in topoIIα at 1259–1296 [20]. In topoIIβ nuclear localisation signals have been found in the C-terminal domain, with two strong NLSs have been identified at 1522–1548 and 1538–1573, with a weaker sequence at 1294–1332 [20], [22]. Studies with isolated topoIIα and β C-terminal domains tagged with Yellow Fluorescent Protein showed that the two were differently localised in the nucleus [23].

In human topoIIα an NES was initially localised to the region 1018–1088 [24], and subsequently this was narrowed down to two sequences, 1017–1028 and 1054–1066, the latter of which is the stronger sequence [25]. In topoIIβ an NES sequence has been idenfied between residues 1034–1044 [24].

Work with chimeric ‘tail swap’ proteins, where the C-terminal domain of topoIIα or topoIIβ is joined to the main body of the enzyme belonging to the opposite isoform, has been reported. A chimera of murine topoII consisting of the body of topoIIα and the tail of topoIIβ showed that this protein was unable to support growth [26]. Conversely, a study examining the ability of human topoII chimeric tail swap proteins to rescue topoIIα cells *in vivo* found that the chimeric proteins, particularly those bearing the topoIIα C-terminal domain, could support growth [27].

To assess whether the C-terminal domain, as the most diverse part of the protein, impacted the relative activities of topoIIα and β, we aimed to construct and characterise *in vitro* and *in vivo* full length and C-terminally truncated forms of human topoIIα and β, and two ‘tail swap’ chimeric proteins where the C-terminal domain of each isoform is linked to the main sequence of the opposite isoform. In contrast to a recently published study describing the *in vivo* characterisation of tail swap proteins, where the C-terminal domain boundary was determined by alignment [27], the constructs described here have boundaries chosen based on those determined by limited proteolysis [28]. The construction process, as well as subsequent characterisation, is reported here.

## Materials and Methods

### Reagents

All chemicals were purchased from Sigma, BDH or Boehringer Mannheim. Restriction enzymes were purchased from NBL Gene Sciences Ltd, New England Biolabs, or Pharmacia Biotech. T4 Ligase was purchased from Gibco BRL. Etoposide was a gift from Prof. H. Newell, NICR, Newcastle, UK. mAMSA, Merbarone and Suramin were obtained from the Drug Synthesis and Chemistry branch, NCI, Bethesda, MD. Quercetin, Quercetagetin, Myricetin and Baicalein were provided by Prof. L.M. Fisher. mAMCA, DACA and Cl-DACA were provided by Prof. B Baguley, Auckland Cancer Society, New Zealand. All other cytotoxics were purchased from Sigma.

### Plasmids and Yeast Strains

*S. cerevisiae* strain JEL1 was used for overexpression of proteins. Yeast strain JN394*t2-4*, a temperature sensitive strain that is viable at 25°C but non-viable at 35°, was used in complementation analysis.

All of the plasmids encoding topoII isoforms express protein under the control of the GAL1 promoter, and have a URA3 marker gene, the yeast 2μ plasmid replication origin and the β-lactamase gene and replication origin of *E. coli* pBR322 are also present. Plasmid YEpWob6, used to express recombinant human topoIIα, encodes the first 5 amino acids of *S. cerevisiae* topoII fused to residues 29–1531 of human topoIIα. Plasmid YEphTOP2β encodes recombinant human topoIIβ with the S165R mutation, with residues 46–1621 fused to the first 5 amino acids of *S. cerevisiae* topoII [29]–[30]. Plasmid YEphTOP2βKLM encodes recombinant wild type topoIIβ residues 46–1621 (without mutation S165R) fused to the first 5 of *S. cerevisiae* topoII. Plasmid intermediates used in the cloning process to construct C-terminal truncations and tail swaps are described in the ‘Results’ section and figure legends.

### Construction of mutant plasmids

Plasmids encoding truncated topoIIα and topoIIβ, as well as two ‘tail swap’ chimeric proteins with the opposite C-terminal domain fused to the main coding sequence were constructed as described in ‘Results’. In all cases restriction digests were carried out according to manufacturer's instructions. Fragments were separated by agarose gel electrophoresis and then purified using a QIAquick Gel extraction spin column. Ligations were then carried out using T4 ligase and the manufacturer's buffers, incubating with 0.5mM ATP for 16 hours at 4°C.

Tail swap mutants were constructed with triple cloning procedures, then the junction sites were confirmed by sequencing both strands with dideoxy DNA sequencing using appropriate primers and a Sequenase version 2.0 DNA sequencing kit (Amersham).

### Preparation of protein

Recombinant human topoIIα and β proteins were expressed and purified as described previously [28], [31]. ATP dependent and independent relaxation assays were done with purified fractions to identify those free of topoI activity.

### *In vitro* assays

Decatenation assays and cleavage assays with an end-labelled 4.3 kb linear DNA fragment from pBR322 were done as described previously [29]–[30].

### *In vivo* assays

Complementation assays were carried out in a temperature sensitive yeast strain JN394*t2-4*, and plasmids encoding topoIIα and topoIIβ (WT and S165R) full length, C-terminally truncated or chimeric proteins. Yeast were grown in Ura- selective media at the permissive temperature (25°C) to an OD_600_ of 1, and then serially diluted in sterile microtitre trays. These cultures were then transferred to plates with an aluminium replicator, then incubated at the permissive, semi-permissive and non-permissive (25°C, 30°C or 35°C) temperatures respectively on glucose containing media, then growth was scored.

## Results

### Construction of plasmids

**Figure 1:**
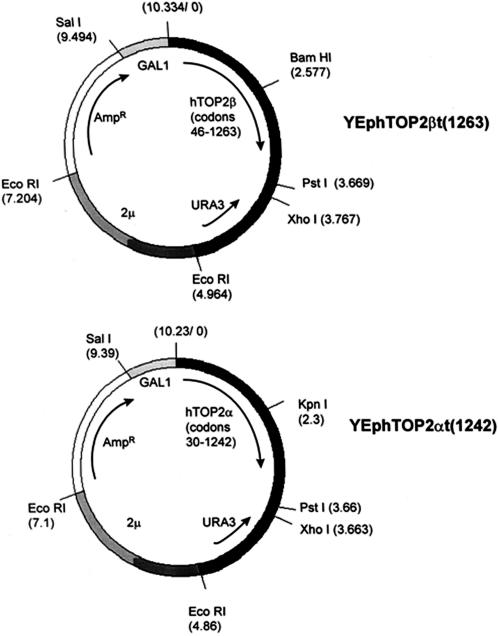
Schematic of C-terminally truncated topoIIα and topoIIβ constructs. Shown are the topoII sequence boundaries, the GAL1 promoter, the 2μ replication origin, the URA3 marker gene, and an ampicillin resistance gene. Restriction sites used in plasmid construction are indicated.

Truncations at the 3′ end of the coding sequence of topoIIα and topoIIβ (S165R) were constructed from plasmids YEpWob6 and YEphTOP2β respectively. The truncated topoIIα plasmid encodes residues 29–1242 and the truncated topoIIβ plasmid encodes residues 46–1263, these being the start of the C-terminal domains as determined by limited proteolysis experiments [28], [32], [33]. Schematics of these plasmids are shown in Figure 1.

In the construction of C-terminally truncated topoIIα, PCR was used to introduce a PstI restriction site in the human topoIIα coding sequence and then to generate the full coding sequence. A fragment was generated between codon 791 (over a KpnI site) and codon 1244 (over the PstI site introduced above) of topoIIα. The PCR product was cloned into a Bluescript plasmid with a XhoI site immediately 3′ to the 792–1242 fragment, then excised by digestion with KpnI and XhoI. This was then cloned into the YEpWob6 plasmid, replacing the fragment 792–1531.

In the construction of C-terminally truncated topoIIβ, multiple internal restriction sites in the topoIIβ sequence meant that a complex cloning procedure was necessary. A fragment containing topoIIβ residues 900–1263 was excised and cloned into a Bluescript plasmid between BamHI and PstI restriction sites, this having a XhoI site 36 residues downstream of the PstI site. A fragment between BamHI to the XhoI beginning at codon 900 was excised and cloned into a vector containing topoIIβ codons 46–899 plus the YEp backbone.

**Figure 2:**
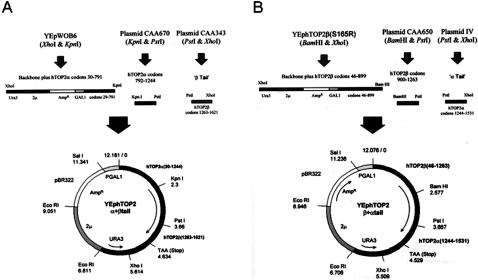
Construction of chimeric ‘tail-swap’ plasmids. A–construction of topoIIα+β tail. TopoIIα fragments 30–791 and 792–1244 and topoIIβ fragment 1263–1621 were generated using restriction digests as shown, then ligated to give the final construct shown. B–construction of topoIIβ+α tail. TopoIIβ fragments 46–899 and 900–1263 and topoIIα fragment 1244–1531 were generated with restriction digests as indicated, then ligated to give the final construct shown [32].

Plasmids encoding chimeric ‘tail-swap’ proteins were created using a triple ligation approach and PCR to generate unique sites. Construction of the topoIIα+β tail plasmid, encoding topoIIα residues 30–1244 fused to topoIIβ residues 1263–1621, is illustrated in figure 2A. Likewise, construction of the topoIIβ (S165R)+α tail plasmid, encoding topoIIβ residues 46–1263 fused to topoIIα residues 1244–1531, is illustrated in figure 2B.

All topoIIβ constructs containing the S165R mutation were changed to give wild type sequence by site directed mutagenesis using a Chameleon kit (stratagene) according to manufacturer's instructions.

### TopoII protein activities

**Figure 3:**
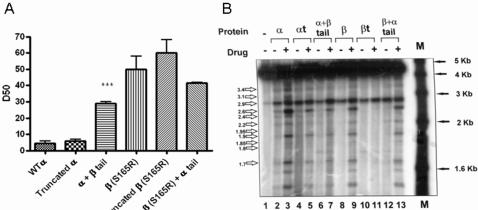
Activity of recombinant proteins. A: Decatenation activity of all proteins, each column is the mean of at least two independent experiments. Standard errors are shown, with significant difference from full length enzyme marker with ‘***’. B: Representative cleavage experiment with 4.3 kb linearised pBR322 DNA with all proteins in the presence of mitoxantrone. TopoIIβ proteins in this case carry the S165R mutation.

The decatenation activity of wild type topoIIα, C-terminally truncated topoIIα, and topoIIα+β tail was assayed. The values for 50% decatenation (D_50_), in ng of protein, are shown in figure 3A. There is no significant difference in D_50_ between topoIIα and its C-terminal truncation, with values of 4.5±1.3 and 6±1 ng of protein respectively. The topoIIα+β tail chimera however does show a significant reduction in decatenation as compared to full length topoIIα, with a D_50_ of 29±1 (p = 0.0008 in a two-tailed unpaired student t-test). This implies that while topoIIα can function perfectly well without a C-terminal domain, the β-tail on the topoIIα enzyme impedes activity.

It is possible that the observed reduction in catalytic activity seen with the topoIIα+β tail chimeric protein was an artefact, caused by the insertion of the tail into the enzyme altering a property such as conformation. A chimeric protein of topoIIβ mutant S165R, known to give a 5-fold reduction in decatenation activity [34], fused to the α-tail, was used to address this concern. If the insertion of the tail *per se* impedes catalytic activity, then the topoIIβ (S165R)+α tail would be expected to have still lower decatenation activity than topoIIβ(S165R). As shown in figure 3A the presence of the α tail gave no reduction in the decatenation activity of topoIIβ(S165R). As with topoIIα, removing the topoIIβ C-terminal domain gave no significant difference in activity, with D_50_ values of 50±8 and 60±8 ng protein for topoIIβ(S165R) and C-terminally truncated topoIIβ(S165R) respectively. The activity of the topoIIβ(S165R)+α tail chimera was increased very slightly with a D_50_ of 41.5±0.5 ng protein, however this difference was not statistically significant (p = 0.4001, figure 3A).

### Complementation analysis of isoforms

To assess the *in vivo* functional activity of the truncated and chimeric proteins, complementation experiments were carried out in the temperature sensitive yeast strain JN394*t2-4*. Data are shown in table 1. All of the topoIIβ (S165R) plasmids were unable to complement at the restrictive temperature, consistent with previous results [34]. Both wild type topoII isoforms supported good growth as expected. The truncated topoIIα and topoIIα+β tail proteins were unable to complement, but interestingly the truncated topoIIβ and topoIIβ+α tail proteins were able to support low levels of growth.

**Table 1:** Complementation of topoII isoforms

	25°C	30°C	35°C
TopoIIα	++	++	++
TopoIIα truncated	+++	+++	-
TopoIIα+β tail	++	++	-
TopoIIβ	++	++	++
TopoIIβ(S165R)	++	++	-
TopoIIβ truncated	++	++	+/−
TopoIIβ(S165R) truncated	++	+	-
TopoIIβ+α tail	++	++	+/−
TopoIIβ(S165R)+α tail	++	++	-

- no growth, +/− poor growth,+some growth, ++ good growth, +++ excellent growth

All experiments were repeated at least twice.

### Cleavage assays with tail swaps

The cleavage activity of each of the wild type and C-terminal truncation mutant proteins was assessed in an end-labelled cleavage assay, in the presence and absence of drug. While the topoIIβ proteins here had mutation S165R this has been shown to have no effect on cleavage under the conditions used [34]. In the absence of drug all six proteins gave cleavage at similar sites (data not shown). While topoIIα gave cleavage at more sites than topoIIβ this difference wasn't statistically significant. The same pattern was seen with the truncated and tail swap proteins, with topoIIα proteins generally giving slightly more cleavage than their topoIIβ counterpart, however this difference wasn't significant.

Drug stimulated cleavage was assayed with flavonoids quercetin, quercetagetin, myricetin, and baicalein, acridines mAMSA and mAMCA, etoposide and mitoxantrone. No difference in cleavage pattern between proteins was seen with drugs with the exception of truncated topoIIβ which promoted cleavage with mAMCA sites corresponding to a combination of topoIIβ and topoIIα. Additionally truncated topoIIβ promoted no cleavage with mitoxantrone (figure 3B), and topoIIα+β tail promoted cleavage with quercetagetin at sites more in common with topoIIβ than topoIIα. This indicates that the C-terminal domain of topoIIβ has a role in the determination of cleavage sites with certain drugs.

## Discussion

Human topoII isoforms α and β, while enzymatically similar *in vitro*, have been shown to have different cellular roles. While topoIIα is thought to be the isoform primarily responsible for DNA segregation, topoIIβ has recently been linked to transcription initiation [2]–[4]. Here we report the construction and characterisation of recombinant truncated and tail swap chimeric proteins. Whilst truncated human topoIIα has been reported previously, the point of truncation in this case was chosen to align with a viral topoII lacking the C-terminal domain [35]. C-terminal truncations of topoIIα and β reported here were based on domain organisation as determined by the cleavage sites in limited proteolysis studies [28]. Likewise, human chimeric tail swap proteins have been described and their *in vivo* function reported, but these also had domain boundaries based on alignment rather than proteolysis sites [27]. Here we report, for the first time, the creation of C-terminally truncated recombinant human topoII proteins based on domain structure indicated by limited proteolysis experiments. Furthermore, we have also created chimeric recombinant human topoII tail swap proteins based on this definition of the C-terminal domain. Additionally, this manuscript is the first report of characterisation of the *in vitro* function of human chimeric tail swap proteins.

Complementation analysis showed, as previously reported, that the S165R mutant proteins were not functional *in vivo* [34]. The topoIIα C-terminal truncation couldn't support growth, in accordance with previous work showing that the loss of the C-terminal domain, and the localisation signals within it, are detrimental to growth [26], [36]. The topoIIα+β chimera was also unable to support growth suggesting that the β C-terminal domain is unable to restore the localisation of the enzyme, or perhaps that the topoIIβ C-terminal domain has a different function to the topoIIα C-terminal domain. This would be consistent with previous experiments showing that human topoIIα preferentially relaxes positive supercoils, whereas topoIIβ showed no preference [37]. This result is in contrast to a study with a murine topoIIα+β protein which was able to support growth in *S. cerevisiae* strain NAY113 [26]. This difference could be species specific, or due to differences in the definition of the start of the C-terminal domain. In the murine study the last 444 amino acids of the β-tail were used to replace the equivalent region on topoIIα, in contrast to 358 residues here, with 356 amino acids of the topoIIα tail lost in the murine chimera in contrast to 289 here.

**Figure 4:**
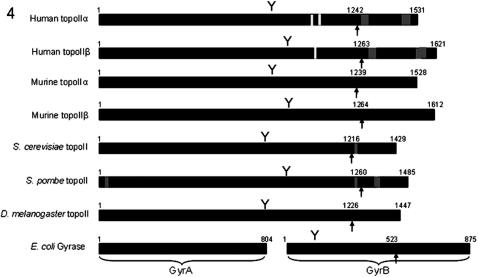
Schematic showing the position of the C-terminal domain of type II topoisomerases. Above each bar are the residue numbers at the start and end of the primary sequence, plus the point equating to the start of the C-terminal domain (indicated by arrows), as determined by limited proteolysis where known, and by alignment with this point where this is not known. Also shown are active site tyrosines (Y), known nuclear localisation sequences (NLS-dark grey) and known nuclear export sequences (NES–light grey). NLS sequences have been identified in human topoIIα (1259–1296, 1454–1497 [20]), human topoIIβ (1294–1332, 1522–1548, 1538–1573 [20]), *S. cerevisiae* topoII (1227–1242 [38]) and *S. pombe* (26–44, 1227–1242, 1322–1339, 1335–1357 [39]). NES sequences have been identified in human topoIIα (1017–1028, 1054–1066 [24]) and human topoIIβ (1034–1044 [24]).

Perhaps more surprising is that topoIIβ truncated protein and topoIIβ+α tail protein can support low levels of growth, implying that some localisation to the nucleus is still present. Known nuclear localisation signals are shown in figure 4 and, with the exception of *S. pombe* topoII which also has an N-terminal signal, all sequences are found in the C-terminal domain [20], [22], [38], [39]. It is therefore unclear why the truncated topoIIβ protein is able to support low levels of growth (and hence localise, albeit inefficiently, to the nucleus), but it is possible that this is due to a presently unknown mechanism, perhaps linked to topoIIβ specific modification, or an unidentified NLS specific to topoIIβ. This would be consistent with previous work that showed that the topoIIα and β C-terminal domains were differently localised [23].

A previous study into human chimeric enzymes found that proliferation of topoIIα knockout human cells was supported in all cases by enzymes bearing the topoIIα C-terminal domain, but that proliferation was only supported rarely and when protein was expressed in large quantities for enzymes bearing the topoIIβ C-terminal domain. The relative levels of growth support are consistent with the data presented here (where the topoIIα C-terminal domain chimera supports low levels of growth and the topoIIβ C-terminal domain chimera supports no growth at all), although the levels of growth differ, perhaps because of differences in the experimental systems or the sensitivity of methods [27].

The decatenation data imply that the topoII C-terminal domain is involved in the modulation of catalytic activity in the two human isoforms. The truncated topoII proteins had no difference in *in vitro* decatenation activity as compared to their full length counterparts suggesting that the C-terminal domain is not necessary for *in vitro* activity, which is consistent with previous data [26], [40], [41]. While the absence of the C-terminal domain for topoIIα or β had no effect on strand passage activity, the presence of the C-terminal domain from the opposite isoform had a noticeable effect with a clear trend emerging. The presence of the β-tail on the topoIIα isoform core gave a statistically significant decrease in strand passage activity compared to the native topoIIα protein, and the presence of the α-tail on the topoIIβ isoform core gave an increase in activity compared to the native topoIIβ protein, although this time not significant, towards that of the native topoIIα protein. As the truncated forms of each protein had no difference in activity when compared to the full length, this implies that it is the presence of the α or β tail that is important for the level of strand passage, acting as a regulator. In this case of the topoIIβ C-terminal domain particularly, this regulation (negative in this case), is quite striking. While it can't formally be excluded that the reduced activity of the chimeric topoIIα+β tail protein is due to the tail swap process, this seems unlikely, as if the process itself reduced activity this should also be seen with the topoIIβ(S165R)+α tail protein. In fact the opposite is seen, with the absence of the β-CTD seeming to ‘release’ the enzyme activity a little and increase the rate of decatenation.

The regulation of catalytic activity by the C-terminal domain could be mediated via differential modification, for instance phosphorylation or SUMOylation, or could be linked to the extensive differences in primary sequence between the two C-terminal domains.

Observations reported previously support the hypothesis that topoIIα and β C-terminal domains are important in differential regulation of the isoforms. All of the SUMO modification sites identified to date have been located in the C-terminal domain of topoII [14]. SUMO conjugation to topoI, topoIIα and topoIIβ has also been linked to the human cellular response to DNA damage [16]–[17]. However, differential degradation of topoIIβ but not topoIIα was observed in response to treatment with ICRF-193, strongly suggesting that the two isoforms are regulated differently by SUMO modification [18].

An analysis of the theoretical protein parameters of full length and truncated topoIIα and topoIIβ, and their tail swap derivatives, is shown in table 2. What is immediately obvious is that, while the full length and truncated topoII isoforms all have similar theoretical pIs, with full length topoIIβ having a slightly lower theoretical pI, the theoretical pI of the isolated C-terminal domain of topoIIβ is considerably lower than other topoII proteins. This is linked to the higher number of acidic residues compared to basic residues seen with this fragment. Unsurprisingly, the α+β tail protein has a lower theoretical pI than full length topoIIα, and the β+α tail protein has a higher theoretical pI than the full length topoIIβ protein, but a similar pI to the truncated topoIIβ protein [42]. It would thus appear that the lower theoretical pI of the β-CTD acts to lower the pI of the core protein to which it is attached. Conversely, the addition of the α-CTD has little effect on either core protein's theoretical pI.

**Table 2:** Protein parameters for human topoII isoforms

	Amino acids	Theoretical pI	Acidic amino acids	Basic amino acids
TopoIIα	1–1531	8.82	226 (14%)	246 (16%)
TopoIIβ	1–1621	8.22	243 (15%)	250 (15%)
Truncated topoIIα	1–1242	8.71	173 (14%)	187 (15%)
Truncated topoIIβ	1–1263	8.83	163 (13%)	185 (15%)
α CTD	1243–1531	9.09	53 (18%)	59 (20%)
β CTD	1264–1621	5.04	77 (22%)	65 (18%)
TopoIIα +β tail	1–1600	7.69	250 (16%)	252 (16%)
TopoIIβ+α tail	1–1552	8.9	219 (14%)	244 (16%)

Shown are the residues of the protein, the theoretical pI and number of acidic (negatively charged, D,E) and basic (positively charged, R,K) amino acids. Shown in parentheses is the percentage of amino acids with each charge in each protein. Reproduced and modified from KL Gilroy, thesis [42].

Cleavage data suggests that the C-terminal domain is not generally involved in cleavage, although there was a consistent, yet non-significant, increase in cleavage with the topoIIα derived proteins. Most drugs showed no difference in drug-stimulated cleavage patterns between topoIIα and β, implying that the C-terminal domain has no impact on the action of these drugs, consistent with previous work showing that topoIIα and topoIIβ cleave at similar sites [43]. There were exceptions to this rule however, for example the truncated topoIIβ showed no cleavage with mitoxantrone, showing that some drugs may have specific interactions that involve the C-terminal domain of human topoIIβ.

In summary, we report the construction of C-terminally truncated and chimeric human topoII enzymes, and show that the C-terminal domain impacts on the activity of the two human isoforms. Further characterisation of human topoIIα and topoIIβ, perhaps by investigating the effect of SUMOylation on either isoform, or the cellular localisation of these tail swap proteins, will be needed to elucidate their different interactions with DNA substrates and functional roles in cells.
